# Physical Activity and Diet Quality Modify the Association between Comorbidity and Disability among Stroke Patients

**DOI:** 10.3390/nu13051641

**Published:** 2021-05-13

**Authors:** Lien T. K. Nguyen, Binh N. Do, Dinh N. Vu, Khue M. Pham, Manh-Tan Vu, Hoang C. Nguyen, Tuan V. Tran, Hoang P. Le, Thao T. P. Nguyen, Quan M. Nguyen, Cuong Q. Tran, Kien T. Nguyen, Shwu-Huey Yang, Jane C.-J. Chao, Tuyen Van Duong

**Affiliations:** 1 Rehabilitation Department, Hanoi Medical University, Hanoi 115-20, Vietnam; lienrehab@hmu.edu.vn; 2 Rehabilitation Center, Bach Mai Hospital, Hanoi 115-19, Vietnam; 3 Rehabilitation Department, Viet Duc University Hospital, Hanoi 110-17, Vietnam; 4 Department of Infectious Diseases, Vietnam Military Medical University, Hanoi 121-08, Vietnam; nhubinh.do@vmmu.edu.vn; 5 Division of Military Science, Military Hospital 103, Hanoi 121-08, Vietnam; 6 Director Office, Military Hospital 103, Hanoi 121-08, Vietnam; vunhatdinh@vmmu.edu.vn; 7 Department of Trauma and Orthopedic Surgery, Vietnam Military Medical University, Hanoi 121-08, Vietnam; 8 Faculty of Public Health, Hai Phong University of Medicine and Pharmacy, Hai Phong 042-12, Vietnam; pmkhue@hpmu.edu.vn; 9 President Office, Hai Phong University of Medicine and Pharmacy, Hai Phong 042-12, Vietnam; 10 Department of Internal Medicine, Haiphong University of Medicine and Pharmacy, Hai Phong 042-12, Vietnam; vmtan@hpmu.edu.vn; 11 Cardiovascular Department, Viet Tiep Friendship Hospital, Hai Phong 047-08, Vietnam; 12 Director Office, Thai Nguyen National Hospital, Thai Nguyen City 241-24, Vietnam; nguyenconghoang@tnmc.edu.vn; 13 President Office, Thai Nguyen University of Medicine and Pharmacy, Thai Nguyen City 241-17, Vietnam; 14 Department of Neurology, Thai Nguyen University of Medicine and Pharmacy, Thai Nguyen City 241-17, Vietnam; tranvantuanyktn@gmail.com; 15 Department of Clinical Pharmacy, Thai Nguyen University of Medicine and Pharmacy, Thai Nguyen City 241-17, Vietnam; 16 Department of Internal Medicine, University of Medicine and Pharmacy, Hue University, Thua Thien Hue 491-20, Vietnam; lphoang@huemed-univ.edu.vn; 17 Health Management Training Institute, University of Medicine and Pharmacy, Hue University, Thua Thien Hue 491-20, Vietnam; nguyenthiphuongthao@hueuni.edu.vn; 18 Department of Health Economics, Corvinus University of Budapest, 1093 Budapest, Hungary; 19 Director Office, Thu Duc City Hospital, Ho Chi Minh City 713-11, Vietnam; quan_minhnguyen@yahoo.com; 20 Director Office, Thu Duc City Health Center, Ho Chi Minh City 713-10, Vietnam; quoccuong.mph@gmail.com; 21 Faculty of Health, Mekong University, Vinh Long 852-16, Vietnam; 22 Department of Health Promotion, Faculty of Social and Behavioral Sciences, Hanoi University of Public Health, Hanoi 119-10, Vietnam; ntk1@huph.edu.vn; 23 School of Nutrition and Health Sciences, Taipei Medical University, Taipei 110-31, Taiwan; sherry@tmu.edu.tw (S.-H.Y.); chenjui@tmu.edu.tw (J.C.-J.C.); 24 Nutrition Research Center, Taipei Medical University Hospital, Taipei 110-31, Taiwan; 25 Research Center of Geriatric Nutrition, Taipei Medical University, Taipei 110-31, Taiwan; 26 Master Program in Global Health and Development, College of Public Health, Taipei Medical University, Taipei 110-31, Taiwan

**Keywords:** stroke patient, Charlson Comorbidity Index, World Health Organization Disability Assessment Schedule II, international physical activity questionnaire, Dietary Approaches to Stop Hypertension Quality, health literacy, International Classification of Diseases, health-related behaviors, Vietnam

## Abstract

Background: Comorbidity is common and causes poor stroke outcomes. We aimed to examine the modifying impacts of physical activity (PA) and diet quality on the association between comorbidity and disability in stroke patients. Methods: A cross-sectional study was conducted on 951 stable stroke patients in Vietnam from December 2019 to December 2020. The survey questionnaires were administered to assess patients’ characteristics, clinical parameters (e.g., Charlson Comorbidity Index items), health-related behaviors (e.g., PA using the International Physical Activity Questionnaire- short version), health literacy, diet quality (using the Dietary Approaches to Stop Hypertension Quality (DASH-Q) questionnaire), and disability (using the World Health Organization Disability Assessment Schedule II (WHODAS II)). Linear regression models were used to analyze the associations and interactions. Results: The proportion of comorbidity was 49.9% (475/951). The scores of DASH-Q and WHODAS II were 29.2 ± 11.8, 32.3 ± 13.5, respectively. Patients with comorbidity had a higher score of disability (regression coefficient, B, 8.24; 95% confidence interval, 95%CI, 6.66, 9.83; *p* < 0.001) as compared with those without comorbidity. Patients with comorbidity and higher tertiles of PA (B, −4.65 to −5.48; *p* < 0.05), and a higher DASH-Q score (B, −0.32; *p* < 0.001) had a lower disability score, as compared with those without comorbidity and the lowest tertile of PA, and the lowest score of DASH-Q, respectively. Conclusions: Physical activity and diet quality significantly modified the negative impact of comorbidity on disability in stroke patients. Strategic approaches are required to promote physical activity and healthy diet which further improve stroke rehabilitation outcomes.

## 1. Introduction

Stroke is a major cause of disability and mortality across the globe [1]. In 2013, there were 113 million disability-adjusted life years, and 6.5 million deaths [2]. In Vietnam, the intracerebral hemorrhage stroke prevalence was as high as that in high-income countries [3]. Stroke and its consequences impose a heavy burden on individuals, the healthcare system, and society in Vietnam and the world [4,5,6].

The risk factors of stroke could be attributed to about 90% modifiable risks, including hypertension, obesity, hyperglycemia, hyperlipidemia, and renal dysfunction [6,7]. Comorbid conditions (or modifiable risks) are common in stroke patients [8,9]. They are predictors of hospital stay, costs, and mortality [10], increase the disability levels [11], and worsen functional outcomes after stroke [12].

Multidisciplinary and multilevel prevention strategies were suggested to prevent stroke. Among those, adequate nutrition, salt reduction, and other dietary interventions are effective strategies for primordial and primary prevention [13]. The longitudinal effect of diet quality (assessed by the Dietary Approaches to Stop Hypertension, or DASH diet) on cardiovascular diseases (CVD), including stroke, was summarized in a meta-analysis of prospective studies [14]. The relationship between diet quality and risk of stroke was also found in a previous large cohort study in European countries [15,16], Taiwan [17], and Hong Kong [18]. The key health behaviors (e.g., diet, physical activity) significantly contribute to cardiovascular conditions (including stroke and other heart and circulatory diseases) [7,19].

The roles of health-related behavioral factors (e.g., physical activity and dietary intake) on stroke prevention were investigated [6,7,19]. However, the modification effects of these factors remain to be explored. It is necessary to explore the potential impacts of health-related behaviors which may modify the negative effects of comorbidity on physical function after stroke. Therefore, we aimed to investigate the modifying impacts of physical activity and diet quality on the relationship between comorbidity and disability among stroke patients.

## 2. Materials and Methods

### 2.1. Study Design and Settings

A cross-sectional study design was used to survey stroke patients between December 2019 and December 2020 in four hospitals in the northern area, one hospital in the central area, and one hospital in the southern area of Vietnam.

### 2.2. Sampling and Sample Size

We used the consecutively convenient sampling technique to recruit patients from cardiovascular, neurology, and rehabilitation departments of selected hospitals. Data of 951 patients were collected from Bach Mai Hospital (11 from the cardiovascular department, 131 from the neurology department, 27 from the rehabilitation center), Military Hospital 103 (293 from the stroke department), Viet Tiep Friendship Hospital (197 from the neurology department), Thai Nguyen National Hospital (197 from the neurology department), Hue University Hospital (45 from the cardiovascular department), and Thu Duc District Hospital (50 from the neurology department).

Patients recruited were those aged ≥ 18 years, in a stable stroke condition diagnosed by a neurologist (e.g., a Mini-Mental State Examination score of ≥22), with the ability to respond to questions. Patients excluded were those with aphasia or visual impairment, or those with diseases that affect cognition (e.g., dementia). Stroke or cerebrovascular disease was defined by the 10th revision of the International Classification of Diseases (ICD-10) codes I60–69; including (I60) Subarachnoid hemorrhage; (I61) Intracerebral hemorrhage; (I62) Other non-traumatic intracranial hemorrhages; (I63) Cerebral infarction; (I64) Stroke, not specified as hemorrhage or infarction; (I65) Occlusion and stenosis of pre-cerebral arteries, not resulting in cerebral infarction; (I66) Occlusion and stenosis of cerebral arteries, not resulting in cerebral infarction; (I67) Other cerebrovascular diseases; (I68) Cerebrovascular disorders in diseases classified elsewhere; and (I69) Sequelae of cerebrovascular disease.

### 2.3. Measurements

#### 2.3.1. Patients’ Characteristics

Participants were asked about their age (years), gender (women vs. men), education attainment (illiterate/elementary school level, junior high school level, senior high school level, college/university level and above), marital status (married vs. single or separated/divorced/widowed), occupation (working vs. retired or infirmity), ability to pay for medication (very or fairly difficult vs. very or fairly easy), and their social status level (low vs. middle or high).

#### 2.3.2. Health-Related Behaviors

Patients were asked about their current behaviors, regarding the status of smoking (never vs. ever smoked), drinking alcohol (no vs. yes).

The International Physical Activity Questionnaire short version (IPAQ-SF) was used for assessing physical activity (PA) level [20]. Patients reported their time spent on different PA types over the last seven days. The IPAQ was validated and used in the Vietnamese population [21]. The overall PA score was calculated by multiplying minutes spent on activities at different levels including vigorous, moderate, walking, and sitting by 8.0, 4.0, 3.3, or 1.0, respectively [20]. The metabolic equivalent task scored in minutes per week (MET-min/wk) was used as the measuring unit of PA [22].

#### 2.3.3. Clinical Parameters

Comorbidity was assessed using the Charlson Comorbidity Index (CCI) items [23,24]. The item list consists of (1) myocardial infarction (history, not ECG changes only); (2) congestive heart failure; (3) peripheral disease (includes aortic aneurysm ≥ 6 cm); (4) cerebrovascular disease or stroke; (5) chronic pulmonary disease; (6) diabetes without end-organ damage (excludes diet-controlled alone); (7) depression; (8) diseases treated with anticoagulants; (9) dementia; (10) hemiplegia; (11) diabetes with end-organ damages (retinopathy, neuropathy, nephropathy, or brittle diabetes); (12) moderate or severe renal diseases; (13) tumor without metastasis (exclude if > 5 years from diagnosis), leukemia (acute or chronic) and lymphoma; (14) moderate or severe liver diseases; (15) metastatic solid tumor; and (16) HIV/AIDS. Patients with dementia were excluded. Cerebrovascular disease, although reported in the list, was excluded when calculating the number of chronic conditions. The comorbidity was classified into two groups (none vs. one or more) to facilitate the analysis.

The body mass index (BMI), a measure of body fat, was calculated as weight (kg)/[height (m)]^2^. The stroke occurrence (first ever vs. recurrent) was also assessed.

#### 2.3.4. Health Literacy

Health literacy (HL) was assessed using the short-form questionnaire with 12 items (HLS-SF12). It was validated and used in Asian countries [25,26], including Vietnam [27,28,29,30]. Patients were asked to rate their perceived difficulty of each item based on 4-point Likert scales from 1 = “very difficult” to 4 = “very easy”. The overall score was standardized to an index ranging from 0 to 50 using Formula (1), with a higher score presenting better HL [31]:

Index = (Mean − 1) × (50/3)(1)

where Index is a specific index score calculated, Mean is the mean of 12 items, 1 is the minimal possible value of the mean (leading to a minimum index score of 0), 3 is the range of the mean, and 50 is the chosen maximum HL index score.

#### 2.3.5. Diet Quality

A brief self-reported measure of diet quality questionnaire, or the Dietary Approaches to Stop Hypertension Quality (DASH-Q) questionnaire, was used for assessing diet quality [32]. The DASH-Q consists of 11 items and asks how many days, over the past 7 days, did patients eat the food items. The response options are from 0 to 7. Two researchers translated the questionnaire into the Vietnamese language. An expert panel (five medical doctors, five public health and nutrition professionals) then validated the content and suggested keeping the original response options and scoring. In Table S1, item “drink milk (in a glass, with cereal, or in coffee, tea, or cocoa)” was removed with a factor loading of −0.45 on component 2, and 0.41 on component 3. The items were loaded on three components, which explained 62.76% of the variance. The DASH-Q with 10 items illustrated adequate convergent validity (item-subscale correlation ranges of 0.70–0.80 on factor 1 and component 3, 0.74–0.89 on component 2,), satisfactory reliability (Cronbach’s alpha of 0.74), and no floor or ceiling effects (Table S1). A sum score of DASH-Q was recommended for use in research and clinical practices. The overall DASH-Q scores range from 0 to 70, with higher scores presenting better diet quality. Participants with high diet quality were considered adherent to DASH nutritional recommendations [32].

#### 2.3.6. Disability

The 12-item World Health Organization Disability Assessment Schedule II (named as WHODAS II) was used for assessing the disability level in different cultures and settings [33,34]. Patients were asked to rate on 5-point scales the extent of difficulty of doing the activities in the past 30 days from (1) none to (2) mild, (3) moderate, (4) severe, (5) extreme difficulty or cannot do [35]. The overall WHODAS II score was a sum score of all items, with a higher score reflecting a higher disability level.

### 2.4. Data Collection Procedure

Research assistants (e.g., doctors, nurses, and medical students) firstly received a four-hour training session about data collection, conducted by researchers from each hospital. Doctors in charge selected qualified patients for the study. Research assistants then approached and asked for patients’ voluntary participation. The informed consent form was signed by patients before administering the survey. The face-to-face interviews were conducted at the bedside. Adequate time was given to patients to complete the survey. It took about 30 min to complete a survey for one patient. After the interview, research assistants reviewed the medical records for clinical parameters.

We postponed the data collection during the COVID-19-induced nationwide lockdown in Vietnam from 1st to 22nd of April 2020 [36,37]. During the pandemic, research assistants also received infection control training from each hospital, in terms of mask-wearing, handwashing, and physical distancing which followed the guidelines of the Centers for Disease Control and Prevention (CDC) [38], World Health Organization (WHO) [39], and Ministry of Health in Vietnam [40].

### 2.5. Ethical Consideration

The study was reviewed and approved by the Institutional Ethical Review Committee of Hanoi University of Public Health (IRB No. 498/2019/YTCC-HD3 and No. 312/2020/YTCC-HD3). All subjects gave their informed consent for inclusion before they participated in the study.

### 2.6. Statistical Analysis

Firstly, the studied variables’ distributions were explored using descriptive analysis. A one-way ANOVA test was used to compare the distribution of WHODAS II in different categories of independent variables. Secondly, the associations of comorbidity, physical activity, and diet quality (DASH-Q) with disability (WHODAS II) were analyzed using bivariate and multivariate linear regression models. We adjusted for age, gender, marital status, education, occupation, smoking status, and health literacy in multivariate linear regression models as these variables showed associations with WHODAS II in the bivariate linear regression models (Table S2). Finally, the interaction analysis was performed to examine the potential modification impacts of physical activity and diet quality on the association between comorbidity and disability. To visualize the results of the interaction models, we conducted a simple slope analysis using PROCESS Macro of SPSS for moderation analysis [41]. The slope plots were drawn using the evaluated values of WHODAS II for two categories of comorbidity (non-CCI vs. CCI) by three values of physical activity (mid-tertile 1, mid-tertile 2, and mid-tertile 3 of MET-min/wk), and diet quality (Z = −1, one standard deviation below the mean; Z = 0, the mean; Z = +1, 1 standard deviation above the mean of DASH-Q). Data were analyzed using IBM SPSS Version 20.0 (IBM Corp, Armonk, NY, USA) [42]. The significance level was set at a *p*-value < 0.05.

## 3. Results

### 3.1. Patients’ Characteristics

In the total sample, 70% of stroke patients were 60 years old or above, 59.2% were men. The proportions of patients with comorbid and first-ever stroke were 49.9% and 82.5%, respectively. The scores of DASH-Q, HL, and WHODAS II were 29.2 ± 11.8, 23.4 ± 10.0, 32.3 ± 13.5, respectively. The score of WHODAS II was significantly varied in different categories of age, gender, marital status, education, occupation, comorbidity, smoking, and physical activity (Table 1).

### 3.2. Associations of Comorbidity, Physical Activity, Diet Quality with Disability

The results of the multivariate analysis (after adjusting for age, gender, marital status, education attainment, occupation, smoking status, and health literacy) illustrate that patients with comorbidity had a higher disability score (regression coefficient, B, 8.24; 95% confidence interval, 95%CI, 6.66, 9.83; *p* < 0.001) as compared with those without comorbidity. In comparison with patients’ exercise level in the first tertile, those in the second tertile (B, −6.49; 95%CI, −8.51, −4.47; *p* < 0.001), or third tertile (B −9.00; 95%CI, −11.06, −6.94; *p* < 0.001) had a lower score of disability. Patients with a one-point increment in DASH-Q had a 0.20-point reduction in disability (B, −0.20; 95%CI, −0.27, −0.13; *p* < 0.001; Table 2).

### 3.3. Modification Impacts of Physical Activity, Diet Quality

**Figure 1:**
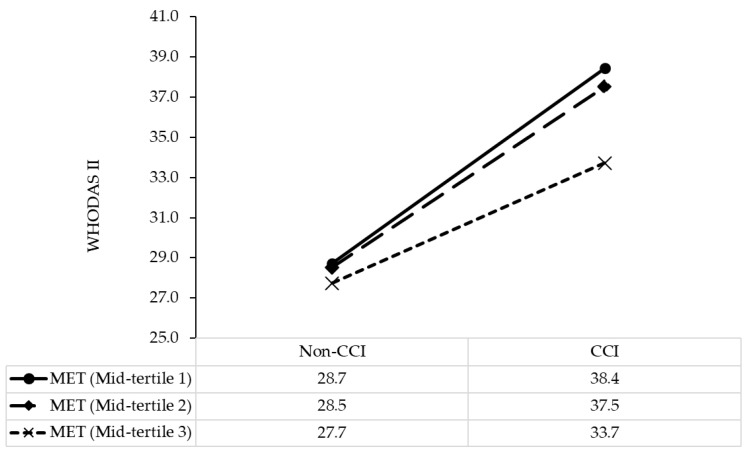
Simple slope plot for the interaction between comorbidity and physical activity on disability among stroke patients (*n* = 951). CCI, Charlson Comorbidity Index; WHODAS II, World Health Organization Disability Assessment Schedule II; MET, metabolic equivalent task scored in minutes per week.

In the multivariate analysis, in comparison with patients with no comorbidity and in the lowest tertile of physical activity (PA), those with a comorbidity and in the lowest tertile of PA had scores of disability 10.67 points higher (B, 10.67; 95%CI, 7.96, 13.37; *p* < 0.001), and those in the second tertile of PA (B, −4.65; 95%CI, −8.44, −0.85; *p* < 0.016), and third tertile of PA (B, −5.48; 95%CI, −9.27, −1.70; *p* < 0.005) had scores of disability 4.65 and 5.48 points lower, respectively (Table 3). The model results are illustrated in Figure 1.

**Figure 2:**
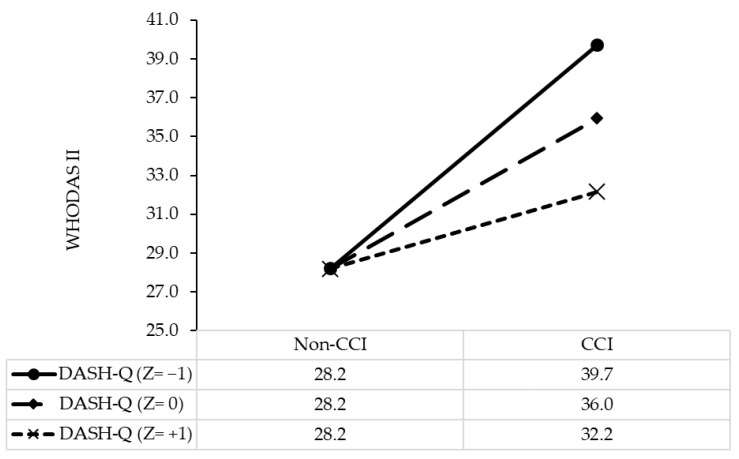
Simple slope plot for the interaction between comorbidity and diet quality on disability among stroke patients (*n* = 951). Note: Z = −1, one standard deviation below the mean; Z = 0, the mean; Z = +1, 1 standard deviation above the mean. CCI, Charlson Comorbidity Index; WHODAS II, World Health Organization Disability Assessment Schedule II; DASH-Q, Dietary Approaches to Stop Hypertension Quality.

Similarly, in comparison with patients with no comorbidity and the lowest score of DASH-Q, those with a comorbidity and the lowest score of DASH-Q had scores of disability 17.12 points higher (B, 17.12; 95%CI, 12.98, 21.45; *p* < 0.001), and those with a one-point increment in DASH-Q had scores of disability 0.32 points lower (B, −0.32; 95%CI, −0.45, −0.19; *p* < 0.001; Table 3). The model results are illustrated in Figure 2.

## 4. Discussion

In this study, comorbidity was found as one of the key predictors of disability in stroke patients. Comorbid medical conditions were strongly associated with poor outcomes and death [43,44,45], with higher disability levels [11], and worse functional outcomes after stroke [12]. It is necessary to evaluate the comorbid conditions in order to help develop appropriate plans for treatment and rehabilitation.

A higher physical activity score was associated with a lower disability score in the current study. Daily physical activity was found to be independently associated with a better physical component of quality of life in stroke patients, in a previous study [46]. Stroke patients spent more time on sedentary behaviors which further affect physical function [47,48,49] and recovery after stroke [50]. The prevalence of physical inactivity was increased during the COVID-19 pandemic which further created a huge burden of cardiovascular disease [51,52]. Therefore, physical activity should be promoted to potentially reduce physical limitation or disability in people living with stroke and improve the outcomes of rehabilitation therapy.

A healthy diet has been found to exert beneficial effects on cardiovascular disease (CVD) prevention [53,54,55]. The diet quality was associated with CVD-free life expectancy [56]. However, the certainty of the evidence was low [53,57]. In our current study, a higher score of diet quality was also associated with a lower disability score. However, a previous study showed no significant role of nutrition therapy in activities of daily living (ADL) in older stroke patients [58]. In a 5.3-year follow-up study conducted on older adults, a healthy diet (e.g., DASH diet) was associated with a lower likelihood of ADL disability and mobility disability [59]. In addition, adherence to a healthy diet was associated with a lower frailty index, reflecting an aspect of disability in older adults [60,61]. Moreover, in the older population, a healthy diet has also shown positive associations with muscle mass, muscle strength, and physical performance [62]. Therefore, it is suggested that a healthy diet may be an effective approach for the prevention of malnutrition and functional disability in older people, especially in those with stroke. A previous study showed that early nutritional intake after acute stroke admission had positive impacts on home discharge and ADL [63].

A healthy diet has been linked with lower concentrations of inflammatory parameters as risk factors for cardiovascular diseases [64]. During the COVID-19 pandemic, nutrition therapy has been found to be a potential protective approach to support the immune system, and reduce inflammation [65], while several nutrients may enhance the functional status [66]. Moreover, patients with better nutritional status had a lower thrombosis incidence which is recognized as a risk of cerebrovascular disease [67], e.g., stroke caused by COVID-19 infection [68]. Equally important to a healthy diet, physical activity has the potential to improve inflammatory status and mobility in chronic stroke patients [69]. Inflammation is common in several chronic diseases [70]. It shows an association with malnutrition, functional outcomes [71], and disability [72].

In the current study, physical activity and diet quality significantly modified the negative impacts of comorbidity on disability in stroke patients. A previous study has shown that being physically active and eating a healthy diet were associated with lower disability-adjusted life years (DALYs), and those who adhere to more healthy behaviors lived longer in good health [73]. During the COVID-19 pandemic, the adherence to a healthy diet slightly increased [74,75], although unhealthy food consumption and physical inactivity also increased [74,76]. Importantly, both a healthy diet and physical activity have shown benefits for first and recurrent stroke prevention [77]. Therefore, promoting a lifelong healthy lifestyle is the most important way to primarily prevent CVD, as emphasized in the updated guidelines for primary CVD prevention [78].

Our study has some limitations. Firstly, the study sample was relatively small, which limits the analysis of the impact of the individual comorbid conditions on disability. Secondly, the duration of different comorbid diseases was not investigated in the current study, which affected the analysis of the association. Thirdly, the potential factors that may render food intake difficult (i.e., dysphagia after stroke, modification of diet texture) were not investigated in our study which might confound the findings. Finally, the generalizability and causality cannot be inferred from a cross-sectional design with consecutive convenient sampling. For example, it is also possible that disability affects food availability (e.g., no access to fresh food, no possibility to cook), which may explain the observed correlation. A longitudinal design is required to confirm the association. Despite the abovementioned limitations, the findings indicate a phenomenon and direction for future research and provide evidence for strategic interventions which may alleviate the negative impact of the comorbid condition on disability in stroke patients.

## 5. Conclusions

In stroke patients, comorbidity, physical activity, and diet quality were significantly associated with disability status. Importantly, the negative impact of comorbidity on disability was modified by physical activity and diet quality. The findings suggest that assessing comorbidity and promoting healthy lifestyles in clinical practice are important to improve stroke rehabilitation outcomes.

## Figures and Tables

**Table 1:** Characteristics and disability in stroke patients (*n* = 951).

Variables	Total	WHODAS II	*p* *
	*n* (%)	(Mean ± SD)	
Age, years			<0.001
19–59	285 (30.0)	30.4 ± 13.1	
60–69	286 (30.1)	31.2 ± 13.5	
70–79	222 (23.3)	33.3 ± 13.4	
80–99	158 (16.6)	36.5 ± 13.3	
Gender			0.035
Women	388 (40.8)	33.5 ± 13.3	
Men	563 (59.2)	31.6 ± 13.5	
Marital status			0.040
Married	837 (88.0)	32.0 ± 13.6	
Single or Widowed/Divorced/Separated	114 (12.0)	34.8 ± 12.4	
Education attainment			0.002
Illiterate or elementary	215 (22.6)	34.4 ± 12.3	
Junior high	257 (27.1)	33.6 ± 14.3	
Senior high	251 (26.4)	31.4 ± 14.5	
College/university or higher	227 (23.9)	30.1 ± 12.0	
Occupation			<0.001
Working	518 (54.5)	29.6 ± 12.9	
Retired or infirmity	433 (45.5)	35.6 ± 13.4	
Ability to pay for medication			0.528
Very or fairly difficult	423 (44.5)	32.7 ± 13.7	
Very or fairly easy	528 (55.5)	32.1 ± 13.3	
Social status			0.344
Low	111 (11.7)	33.5 ± 12.3	
Middle or high	840 (88.3)	32.2 ± 13.6	
BMI, kg/m^2^			0.203
Underweight (<18.5)	90 (9.5)	33.5 ± 14.6	
Normal weight (18.5 ≤ BMI < 24.0)	794 (83.7)	32.4 ± 13.4	
Overweight/obese (BMI ≥ 25.0)	65 (6.8)	29.7 ± 12.8	
CCI			<0.001
None	476 (50.1)	28.1 ± 11.8	
One or more	475 (49.9)	36.6 ± 13.8	
Stroke occurrence			0.261
First ever	785 (82.5)	32.1 ± 13.2	
Recurrent	166 (17.5)	33.4 ± 14.8	
Smoking			0.028
Never smoked	544 (57.2)	31.5 ± 13.5	
Ever smoked	407 (42.8)	33.5 ± 13.4	
Drinking alcohol			0.071
No	661 (69.5)	32.9 ± 13.7	
Yes	290 (30.5)	31.2 ± 13.0	
Physical activity, MET-min/wk			<0.001
Tertile 1 (MET ≤ 597)	324 (34.1)	38.4 ± 13.3	
Tertile 2 (597 < MET ≤ 3726)	312 (32.8)	31.0 ± 13.2	
Tertile 3 (MET > 3726)	315 (33.1)	27.5 ± 11.6	
DASH-Q, mean ± SD	29.2 ± 11.8		
HL index, mean ± SD	23.4 ± 10.0		
WHODAS II, mean ± SD	32.3 ± 13.5		

Abbreviation: SD, standard deviation; WHODAS II, World Health Organization Disability Assessment Schedule II; BMI, body mass index; CCI, Charlson Comorbidity Index; MET-min/wk, metabolic equivalent task scored in minutes per week; DASH-Q, Dietary Approaches to Stop Hypertension Quality; HL, health literacy.* Results of one-way ANOVA test.

**Table 2:** Associations of comorbidity, physical activity, and diet quality with disability among stroke patients (*n* = 951).

Variables	WHODAS II		WHODAS II	
	B (95%CI) *	*p*	B (95%CI) **	*p*
CCI				
None	Reference		Reference	
One or more	8.51 (6.88, 10.14)	<0.001	8.24 (6.66, 9.83)	<0.001
Physical activity, MET-min/wk				
Tertile 1	Reference			
Tertile 2	−7.40 (−9.37, −5.42)	<0.001	−6.49 (−8.51, −4.47)	<0.001
Tertile 3	−10.82 (−12.80, −8.85)	<0.001	−9.00 (−11.06, −6.94)	<0.001
DASH-Q, 1-point increment	−0.27 (−0.34, −0.20)	<0.001	−0.20 (−0.27, −0.13)	<0.001

Abbreviation: WHODAS II, World Health Organization Disability Assessment Schedule II; CCI, Charlson Comorbidity Index; MET-min/wk, metabolic equivalent task scored in minutes per week; DASH-Q, Dietary Approaches to Stop Hypertension Quality.* Results of bivariate linear regression analysis.** Results of multivariate linear regression analysis after adjustment for age, gender, marital status, education attainment, occupation, smoking status, and health literacy.

**Table 3:** Interactions of comorbidity with physical activity and diet quality on disability among stroke patients (*n* = 951).

Interactions	WHODAS II		WHODAS II	
	B (95%CI) *	*p*	B (95%CI) **	*p*
CCI and MET				
Non-CCI × MET (tertile 1)	Reference		Reference	
CCI × MET (tertile 1)	10.70 (7.93, 13.47)	<0.001	10.67 (7.96, 13.37)	<0.001
Non-CCI × MET (tertile 2)	−2.74 (−5.59, 0.11)	0.059	−2.50 (−5.31, 0.32)	0.082
Non-CCI × MET (tertile 3)	−6.23 (−9.08, −3.39)	<0.001	−4.56 (−7.40, −1.72)	0.002
CCI × MET (tertile 2)	−5.47 (−9.37, −1.58)	0.006	−4.65 (−8.44, −0.85)	0.016
CCI × MET (tertile 3)	−5.39 (−9.27, −1.50)	0.007	−5.48 (−9.27, −1.70)	0.005
CCI and DASH-Q				
Non-CCI × DASH-Q (lowest score)				
CCI × DASH-Q (lowest score)	18.62 (14.36, 22.88)	<0.001	17.12 (12.98, 21.45)	<0.001
Non-CCI × DASH-Q (1-point increment)	−0.03 (−0.13, 0.07)	0.523	−0.01 (−0.09, 0.09)	0.990
CCI × DASH-Q (1-point increment)	−0.37 (−0.51, −0.24)	<0.001	−0.32 (−0.45, −0.19)	<0.001

Abbreviations: WHODAS II, World Health Organization Disability Assessment Schedule II; CCI, Charlson Comorbidity Index; MET, metabolic equivalent task scored in minutes per week; DASH-Q, Dietary Approaches to Stop Hypertension Quality.* Results of bivariate linear regression analysis.** Results of multivariate linear regression analysis adjusted for age, gender, marital status, education, occupation, smoking status, and health literacy.

## Data Availability

Data will be available on the reasonable request from the corresponding author.

## Supplementary Materials

The following are available online at https://www.mdpi.com/article/10.3390/nu13051641/s1, Table S1: Construct and convergent validity, internal consistency, floor and ceiling effects the healthy eating score (*n* = 951); Table S2: Associated factors of disability via bivariate linear regression analysis (*n* = 951).
