# Supplementary material for: Physical Activity and Diet Quality Modify the Association between Comorbidity and Disability among Stroke Patients
Source: Nutrients. 2021 May 13;13(5):1641. doi: 10.3390/nu13051641 (PMC8152968; doi:10.3390/nu13051641)
Supplement: Supplementary file 1 [file nutrients-13-01641-s001.zip › nutrients-1203980-supplementary.pdf]

Table S1. Construct and convergent validity, internal consistency, floor and ceiling effects the healthy eating score (*n*=951).

	DASH-Q		
Factor loading value [*]	Component 1	Component 2	Component 3
1. Nuts or peanut butter	0.67		
2. Beans, peas, or lentils	0.72		
3. Eggs	0.71		
4. Pickles, olives, or other vegetables in brine	0.78		
5. Fruits and vegetables (≥5 servings)		0.75	
6. Fruits (>1 serving)		0.86	
7. Vegetables (>1 serving)		0.81	
8. Broccoli, collard greens, spinach, potatoes, squash, or sweet potatoes			0.82
9. Apples, bananas, oranges, melon, or raisins			0.70
10. Whole-grain breads, cereals, grits, oatmeal, or brown rice			0.75
Percentage of variance, %		62.76	
Item-subscale convergent validity, mean of Rho (range)	0.74 (0.70-0.80)	0.83 (0.74-0.89)	0.76 (0.70-0.80)
Item-overall scale convergent validity, mean of Rho (range)		0.56 (0.40-0.73)	
Internal consistency, Cronbach's alpha		0.74	
Floor effects, %		0.70	
Ceiling effect, %		0.00	

Abbreviation: DASH-Q, dietary approaches to stop hypertension quality; Rho, Spearman's correlation coefficient.
[*] Principal component analysis using Promax rotation method. The item removed is "drink milk (in a glass, with cereal, or in coffee, tea, or cocoa)" with factor loading of -0.45 on component 2, and 0.41 on component 3 in the initial analysis.

Table S2. Associated factors of disability via bivariate linear regression analysis (*n*=951).

	WHODAS II	
	B (95%CI)*	p
Age, year		
19-59		
60-69	0.83 (-1.36, 3.02)	0.455
70-79	2.91 (0.57, 5.25)	0.015
80-99	6.16 (3.56, 8.75)	<0.001
Gender		
Women		
Men	-1.87 (-3.62, -0.13)	0.035
Marital status		
Married		
Single or Widow/Divorce/Separate	2.76 (0.13, 5.40)	0.04
Education attainment		
Illiterate or elementary		
Junior high	-0.73 (-3.16, 1.69)	0.553
Senior high	-2.98 (-5.42, -0.54)	0.017
College/university or higher	-4.21 (-6.71, -1.71)	0.001
Occupation		
Working		
Retired or infirmity	5.98 (4.30, 7.66)	<0.001
Ability to pay for medication		
Very or fairly difficult		
Very or fairly easy	-0.55 (-2.28, 1.17)	0.528
Social status		
Low		
Middle or high	-1.29 (-3.96, 1.38)	0.344
BMI, kg/m^2		
Under weight (<18.5)	1.04 (-1.90, 3.98)	0.489
Normal weight (18.5 ≤ BMI < 24.0)		
Overweight/obese (BMI ≥ 25.0)	-2.75 (-6.17, 0.66)	0.114
CCI		
None		
One or more	8.51 (6.88, 10.14)	<0.001
Stroke occurrence		
First-ever		
Recurrent	1.30 (-0.96, 3.56)	0.261
Smoking		
Never smoke		
Ever smoke	1.94 (0.21, 3.68)	0.028
Drinking alcohol		
No		

Yes	-1.71 (-3.58, 0.15)	0.071
Physical activity, MET min/wk		
Tertile 1		
Tertile 2	-7.4 (-9.37, -5.42)	<0.001
Tertile 3	-10.82 (-12.80, -8.85)	<0.001
DASH-Q	-0.27 (-0.34, -0.20)	<0.001
HL index	-0.24 (-0.33, -0.16)	<0.001

Abbreviation: WHODAS II, world health organization disability assessment schedule II; BMI, body mass index; CCI, Charlson comorbidity index; MET min/wk, metabolic equivalent task scored in minute per week; DASH-Q, Dietary Approaches to Stop Hypertension quality, HL, health literacy.
